# Monocrystalline Silicon Carbide Disk Resonators on Phononic Crystals with Ultra-Low Dissipation Bulk Acoustic Wave Modes

**DOI:** 10.1038/s41598-019-54278-9

**Published:** 2019-12-10

**Authors:** Benoit Hamelin, Jeremy Yang, Anosh Daruwalla, Haoran Wen, Farrokh Ayazi

**Affiliations:** 1 Department of Electrical and Computer Engineering, Georgia Institute of Technology, 777 Atlantic Drive NW, Atlanta, GA 30332 USA; 2 School of Physics, Georgia Institute of Technology, 837 State Street, Atlanta, GA 30332 USA

**Keywords:** Acoustics, Electrical and electronic engineering, Electronic and spintronic devices

## Abstract

Micromechanical resonators with ultra-low energy dissipation are essential for a wide range of applications, such as navigation in GPS-denied environments. Routinely implemented in silicon (Si), their energy dissipation often reaches the quantum limits of Si, which can be surpassed by using materials with lower intrinsic loss. This paper explores dissipation limits in 4H monocrystalline silicon carbide-on-insulator (4H-SiCOI) mechanical resonators fabricated at wafer-level, and reports on ultra-high quality-factors (*Q*) in gyroscopic-mode disk resonators. The SiC disk resonators are anchored upon an acoustically-engineered Si substrate containing a phononic crystal which suppresses anchor loss and promises *Q*_ANCHOR_ near 1 Billion by design. Operating deep in the adiabatic regime, the bulk acoustic wave (BAW) modes of solid SiC disks are mostly free of bulk thermoelastic damping. Capacitively-transduced SiC BAW disk resonators consistently display gyroscopic m = 3 modes with *Q*-factors above 2 Million (M) at 6.29 MHz, limited by surface TED due to microscale roughness along the disk sidewalls. The surface TED limit is revealed by optical measurements on a SiC disk, with nanoscale smooth sidewalls, exhibiting *Q* = 18 M at 5.3 MHz, corresponding to *f* · *Q* = 9 · 10^13^ Hz, a 5-fold improvement over the Akhiezer limit of Si. Our results pave the path for integrated SiC resonators and resonant gyroscopes with *Q*-factors beyond the reach of Si.

## Introduction

Resonant microelectromechanical systems (MEMS) have permeated a wide range of applications, from consumer electronics to precision robotics and inertial navigation on chip^1^, using at their heart resonators as mass sensors, pressure sensors, clocks, accelerometers and gyroscopes^2^. The resolution of MEMS resonators and resonant gyroscopes in particular is limited by electrical and Brownian mechanical noise^3^, which can be reduced by minimizing energy dissipation to the surroundings^4^. Specifically, the Brownian mechanical noise scales as *Q*^−1/2^ where *Q* or *Q*-factor is inversely proportional to energy dissipation. To achieve the lowest possible Brownian mechanical noise and high signal-to-noise ratio, advanced structural designs have been developed in silicon (Si) resonators to minimize energy dissipation and bind *Q* to intrinsic quantum-based losses combining bulk thermoelastic damping (*Q*_TED_) and Akhiezer damping (*Q*_AKHIEZER_) in the megahertz frequency range^5,6^. For example, energy dissipation in substrate-decoupled gyroscopic-mode silicon (Si) disk resonators approaches the overall quantum limit^7,8^ while TED-free Lamé resonators cooled down to 120 K have reached the Akhiezer limit of Si^9^.

Due to ease of fabrication and wafer-level patterning with nanoscale precision, Si has been the prominent structural material for MEMS resonators and resonant gyroscopes. To further improve *Q* beyond the quantum limits of Si, materials that exhibit lower intrinsic dissipation are under active investigation^3^. Chief among those is monocrystalline silicon carbide (SiC), an appealing substrate due to its record-low Akhiezer damping compared to all other commonly micro-machinable substrates such as diamond^10^ and SiN^11^ and availability of 4” monocrystalline on-axis 4H-SiC wafers. Recent theoretical studies show that Akhiezer damping in SiC can be 30X lower than in Si in the megahertz frequency spectrum^3,12^. Furthermore, SiC electronics can operate at temperatures exceeding 800 °C, drastically improving the near-150 °C limit of Si electronics and conferring high-temperature robustness to an all-SiC MEMS and Electronics approach^13^. An all-SiC platform could demonstrate navigation-grade bulk acoustic wave (BAW) gyroscopes that can be deployed in harsh environments such as underground drilling bits.

The cost of production-grade monocrystalline SiC substrates, the restricted deployment of dedicated dry etchers, and the chemical inertness of monocrystalline SiC have tremendously obstructed the realization of SiC resonators with ultra-low dissipation. Through fabrication of fusion-bonded SiC-on-Insulator (SiCOI) substrates, implementation of a substrate-decoupling approach robust to fabrication inaccuracies and the development of nanoscale-precision deep reactive ion etching (DRIE) of SiCOI substrates, this paper lifts some of these limitations and explores the suitability of thick on-axis monocrystalline 4H-SiCOI substrates for mode-matched resonant BAW disk gyroscopes. Advanced processing techniques critical to circumvent extrinsic losses are successfully demonstrated in SiCOI substrates, including forming polycrystalline silicon plugs and defining phononic crystals in the Si handle layer. For the first time, centrally-supported and completely solid SiC disk resonators demonstrate *f* · *Q* products near 9.5 · 10^13^ Hz, well beyond the reach of Si in the megahertz frequency range.

## Results

### Design of ultra-low dissipation SiC disk resonators with gyroscopic modes

**Figure 1:**
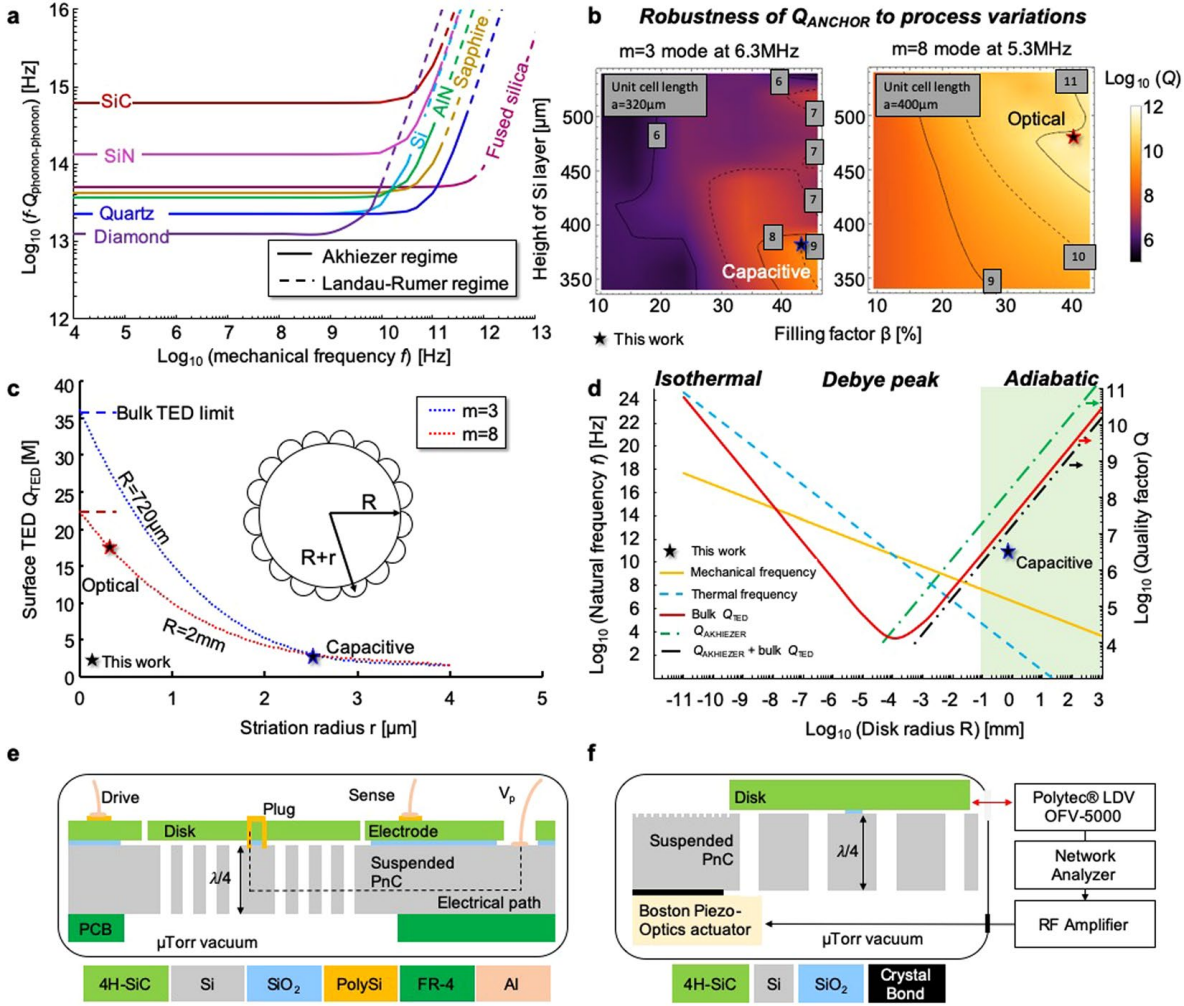
Design elements of SiC micromechanical resonators with ultra-low energy dissipation. (**a**) SiC MEMS resonators with dissipation solely bound by anharmonic phonon-phonon scattering in the Akhiezer regime are poised to exhibit record-high *f* · *Q* products among micro-machinable substrates. (**b**) Numerical simulations reveal the 2.5D substrate-decoupling approach shields *Q*_ANCHOR_ from fabrication and design inaccuracies. (**c**) As-born vertical, closely compact and microscale wide striations along the disk’s sidewall (see the inset schematic) generate surface TED which limits *Q*_TOTAL_ to ~3 M in capacitive disks and to ~18 M in optical disks. (**d**) Bulk TED dominates Akhiezer damping in SiC disk resonators with flush sidewalls and operating the m = 3 modes deep in the adiabatic regime. (**e**) Cross-sectional schematic of a centrally-anchored m = 3 mode capacitive SiC BAW disk resonator; a polySi plug provides an electrical path without requiring any physical contact to the SiC disk. (**f**) Cross-sectional schematic of a centrally-anchored m = 8 mode SiC BAW disk resonator which motion is sensed by a laser Doppler vibrometer (LDV) unit. By operating a higher-order m = 8 mode in a lower surface-to-volume SiC disk with flush sidewalls, the optical disks are more forgiving to anchor loss and surface loss, revealing the true color of surface TED.

Figure 1a reveals that SiC benefits from an extraordinary low phonon-phonon dissipation limit owing in part to its high Debye average velocity and low Grüneisen parameter, which prevent normal and Umklapp scattering with thermal phonons from siphoning energy when the phonon distribution is perturbed from its equilibrium distribution by the strain produced by acoustic waves^14^. These pristine material properties concur to reduce the amplitude of lattice thermal vibrations, limit scattering opportunities and restrict anharmonic phonon-phonon scattering in the Akhiezer regime. In practice, multiple extrinsic dissipation mechanisms overshadow intrinsic losses in SiC micromechanical resonators:

1 $$\frac{1}{{Q}_{TOTAL}}=\frac{1}{{Q}_{AIR}}+\frac{1}{{Q}_{SURFACE}}+\frac{1}{{Q}_{ANCHOR}}+\frac{1}{{Q}_{TED}}+\frac{1}{{Q}_{AKHIEZER}}$$

Equation (1) guides our design approach to suppress extrinsic loss mechanisms and bind dissipation to intrinsic loss mechanisms. The approach consists in removing anchor loss (*Q*_ANCHOR_) using a non-coplanar 2.5 dimensional (2.5 D) substrate decoupling approach without loading *Q*_TED_ and in operating bulk modes under vacuum to elude air damping (*Q*_AIR_). Concentrating the energy far from the dissipative surfaces of BAW resonators and taking advantage of the high Young’s modulus of SiC reduces the contribution of surface effects (*Q*_SURFACE_) to energy dissipation^15,16^.

Although multiple advanced designs enabling complete substrate deafness (*Q*_ANCHOR_ near 1B) have been successfully implemented in center-supported Si BAW resonant gyroscopes^7,8^, these designs cannot be applied in practice to ultra-high *Q* SiC resonators and resonant gyroscopes. Using disks with substrate-decoupling structures^5,17^, instead of completely-solid disks, introduces additional dissipation pathways, severely degrading *Q*_TED_ and negating the benefits of record-high *Q*_AKHIEZER_. Decreasing the ratio of the SiO_2_ pedestal’s diameter to the SiC disk’s diameter to below 5% and increasing the height of the SiO_2_ pedestal to above 200 μm completely circumvent anchor loss while severely compromising structural integrity and resilience to shocks and vibrations. In this work, the anchor loss of centrally-anchored SiC BAW disk resonators is suppressed and both structural integrity and *Q*_TED_ are preserved. Defined in the Si handle layer, an enhanced phononic crystal (PnC)^14,18^ localizes acoustic energy in the SiC disk resonator through the suppression of acoustic waves travelling in any arbitrary direction in Si under the condition their frequencies are sufficiently close to the target disk’s resonance frequency. By placing the substrate-decoupling PnC and the mechanical resonators in separate planes, the acoustically-deaf Si handle layer can support an array of ultra-high *Q* SiC resonators while maintaining the die’s footprint. Navigation-grade inertial measurement units (IMUs) contain multiple ultra-high *Q* gyroscopes, accelerometers and clocks^1^ and will directly benefit from the demonstration of this non-coplanar 2.5D substrate-decoupling approach which is applied to a single gyroscopic-mode disk resonator in this work. The design details of this 2.5 D frequency-dependent decoupling approach have been published elsewhere^14^. Matching the bandgap center frequency of the Si PnC and the resonance frequency of the SiC BAW disk resonator prevents in-plane waves from travelling in the Si handle layer. Similarly, out-of-plane waves travelling in the Si handle layer are suppressed by quarter-wavelength-matching the thickness of the Si handle layer to the resonance frequency. Through quarter-wavelength matching, the thickness and acoustic properties of the handle layer set the optimal target resonance frequency and define the frequency-setting dimensions of the SiC resonators; any frequency mismatch reduces the efficiency of this frequency-dependent decoupling approach and loads *Q*_ANCHOR_ (see Supplementary Note 1). The *Q*_ANCHOR_ of 6.29 MHz m = 3 elliptical modes in 1.4 mm-wide and 55 μm-thick SiC disks on a suspended 380 μm-thick Si honeycomb PnC approaches 1B across all simulated process corners, including thickness variations of the Si handle layer as well as design and fabrication inaccuracies of the PnC (Fig. 1b). Suspending the PnC prevents any physical contact and preserves an effective decoupling approach.

The quarter-wavelength-matching condition for SiCOI wafers with 100s- μm-thick Si handle layer places the optimized resonance frequencies at megahertz range, which is also favorable for achieving high *Q*_TED_ in SiC resonators^19^. Megahertz BAW disk resonators with frequency-setting dimensions in the millimeter range operate in the deep adiabatic regime. Because thermo-mechanical couplings are suppressed in the adiabatic regime, high *Q*_TED_ significantly above 1 M can be reached in mm-scale BAW disk resonators, despite the relatively high coefficient of thermal expansion (CTE) of SiC. Perforations or micro-scale sidewall roughness permit additional thermo-mechanical couplings to severely degrade *Q*_TED_^17^, making a perforation-free design and sidewalls with nanoscale smoothness critical characteristics of micromechanical resonators with ultra-high *Q*_TED_. For example, Fig. 1c shows that microscale sidewall roughness along the sidewall of completely-solid millimeter-wide SiC disks substantially drops *Q*_TED_ from 36 M to 3 M, making surface TED^20^ the main damping mechanism in this work. Figure 1d shows dissipation in solid SiC disk resonators operating in the secondary elliptical mode (m = 3) is bound by Akhiezer (*f* · *Q* = 6 · 10^14^ Hz) and bulk TED (*f* · *Q* = 1.2 · 10^14^ Hz), with a combined theoretical *f* · *Q* limit of 1 · 10^14^ Hz. The theoretical limit of SiC surpasses the intrinsic limits of Si, reached recently by Lamé resonators cooled down at 120 K (*f* · *Q* = 2 · 10^13^ Hz)^9^, revealing the benefits of on-axis 4H-SiC as a substrate for resonators with ultra-low dissipation.

The m = 3 elliptical modes implemented in on-axis 4H-SiCOI substrates are suitable candidates for integrated resonant gyroscopes, owing to the excellent isoelasticity of 4H-SiC^21^. Moreover, a polycrystalline silicon (polySi) plug is introduced to reduce any alignment inaccuracies between the SiC disk and the SiO_2_ pedestal to below 500 nm, preserving critical frequency and dissipation degeneracies of Coriolis-coupled modes^5^. Introducing an n-doped polySi plug obviates directly wire bonding the SiC disk to electrically bias it, avoiding disk tilting and preserving *Q*_ANCHOR_, leaving surface TED as the dominant loss mechanism. The tessellation layout encompasses sufficient area to anchor the electrodes. Figure 1e shows a schematic of the electrically-transduced disk design. Since the presence of electrodes surrounding the SiC disk restricts SEM-based sidewall observations and accurate surface TED numerical simulations, optically-interrogated 100 m-thick SiC disks without electrodes and with relaxed fabrication constraints have been also designed, fabricated and tested (Fig. 1f). Hereinafter, SiC disks with electrodes are referred to as capacitive disks and disks without electrodes as optical disks. To observe the true color of surface TED loss, the optical disks operate the more forgiving m = 8 modes at 5.3 MHz (Fig. 1b).

### Fabrication

**Figure 2:**
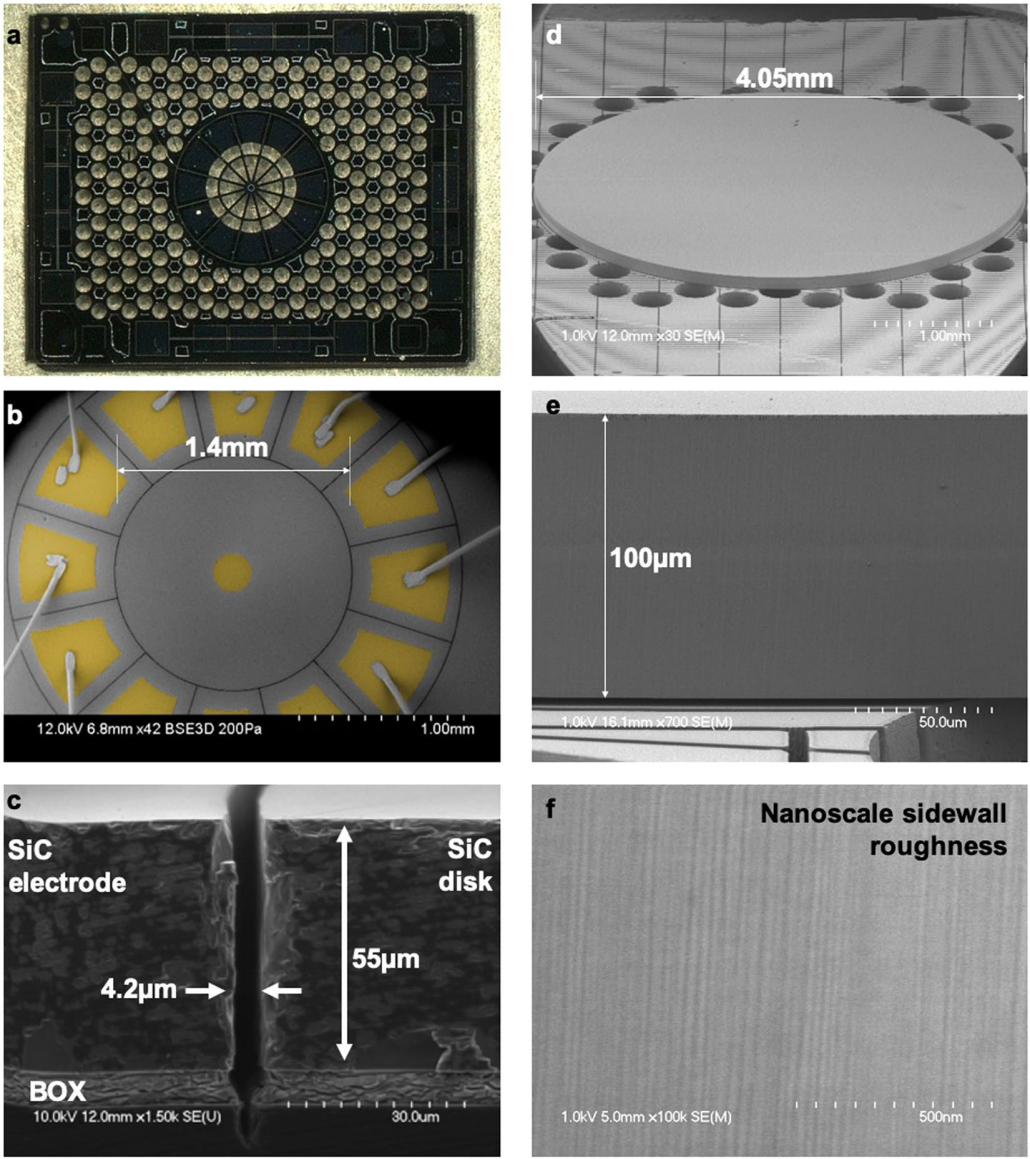
Wafer-level device fabrication on SiCOI substrates with nanoscale sidewall roughness. (**a**) Optical image of a transparent SiC disk and capacitive electrodes, anchored upon a Si phononic crystal with a honeycomb unit cell, spanning nearly the entire 6.5 mm by 8 mm die. (**b**) SEM of the capacitive disk resonator selectively coated with polySi; a polySi plug circumvents needs for wire bonding to the center of the disk (See Supplementary Note 2 for more details). (**c**) Bulk micromachining forms high-aspect-ratio electrostatic transduction gaps in 55 μm-thick SiC. (**d**) With flush sidewalls shown in (**e,f**), optically-interrogated SiC disk resonators reveal very high *Q*s (~18 M) due to low surface TED.

As shown in the Design section, etching SiC trenches with nanoscale roughness is critical in fabricating SiC resonators with dissipation solely bound by Akhiezer damping and bulk TED. Unlike laser machining, dry etching using high-density plasma holds the promise of nanoscale precision micromachining of monocrystalline SiCOI wafers with vertical and smooth sidewall profiles. The main challenge in SiCOI DRIE stems from the chemical inertness of SiC which mandates elevated DC self-bias voltages to generate reasonable etch rates on the order of 300 to 700 nm/min^22,23^. Electroplated Ni is preferred as a hard mask for its 30 to 100:1 selectivity over SiC and for its smooth and vertical sidewall profiles via LIGA patterning which are prerequisites for SiC DRIE with nanoscale precision. Like other metal hard masks, Ni tends to sputter and generates microscale particles that fall into the SiC trenches, locally masking the etch and generating columnar etch defects known as micropillars^24^. The generation of micropillars is disastrous and is bypassed by etching sufficiently narrow trenches at low pressure levels and by flowing an optimized gas composition in the DRIE chamber. Figure 2a–f shows perforation-free solid 4H-SiC disks anchored upon acoustically-deaf Si handle layers with and without surrounding electrodes (see Supplementary Note 2 for fabrication details).

## Characterization

**Figure 3:**
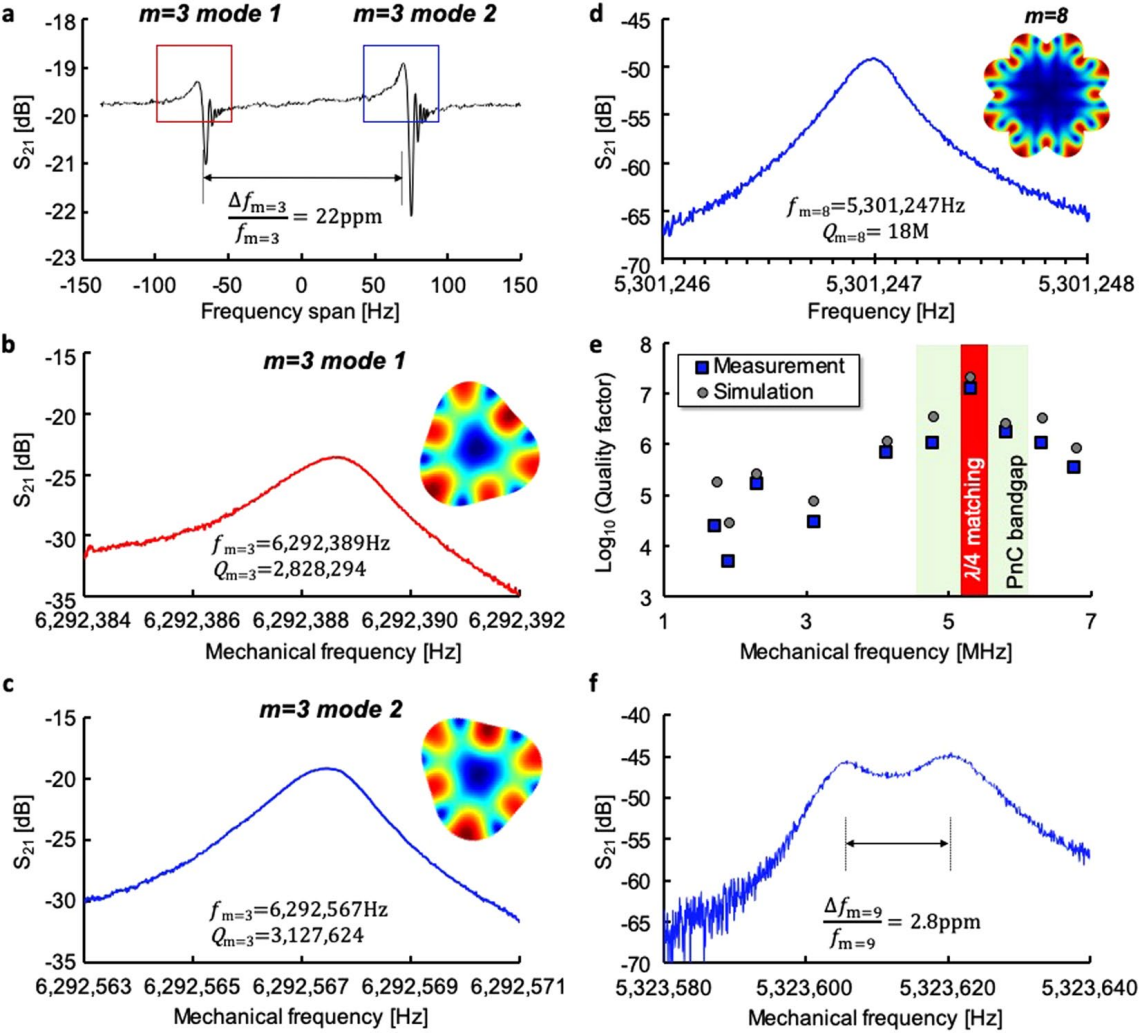
Measurement results from SiC resonators. (**a–c**) The 6.29 MHz gyroscopic m = 3 modes in capacitive disks routinely exhibit *Q*s near 3 M and frequency splits Δ*f*/*f* near 22 ppm. (**d**) The 5.3 MHz m = 8 modes in optical disks with flush sidewalls display *Q*s near 18 M, corresponding to *f* · *Q* = 9.5 · 10^13^ Hz, a record high product for SiC micromechanical resonators in the megahertz frequency range. (**e**) Optical interrogation enables to verify the accuracy of numerical simulations as well as the efficiency of the frequency-dependent 2.5D non-coplanar decoupling approach. (**f**) The optical disks support nearly degenerate frequency splits between m = 9 elliptical modes. For (**a–c**), S_21_ represents the ratio of the input voltage of the Network Analyzer over the output voltage of the Network Analyzer while for (**d&f**), S_21_ represents the ratio of the readout voltage of the LDV over the output voltage of the Network Analyzer.

The details of the apparatus to measure electrostatically-transduced SiC BAW disk resonators are given in the Methods section. Six SiC BAW capacitive disk resonators were fabricated in parallel on a quarter SiCOI wafer and individually tested. The *Q*-factors of the m = 3 modes in these SiC disks are consistently measured above 2 M. Figure 3a–c show the electrostatic measurements of an average capacitive SiC BAW disk resonator with ultra-low energy dissipation. The highest *Q* measured in a capacitive disk is 3.8 M. The m = 3 resonance frequency variations are within ±40 ppm across devices, demonstrating that the frequencies are remarkably robust to process variations. The average m = 3 frequency split is 26 ppm. The smallest measured as-born m = 3 frequency split is 13 ppm. These as-fabricated m = 3 frequency splits result from symmetry-breaking fabrication imperfections and confirm 4H-SiCOI substrates support degenerate Coriolis-coupled secondary elliptical modes in center-supported disk resonators. The 5.5 MHz radial mode is also measured with *Q*s approaching 750 k, lower than the m = 3 modes due to the resonance frequency laying outside the 1.5 MHz-wide PnC bandgap centered on 6.29 MHz. The mode-shape-specific electrode configuration and the relatively large 4.2 μm-wide transduction gap prevent the electrostatic measurement of other in-plane modes.

**Figure 4:**
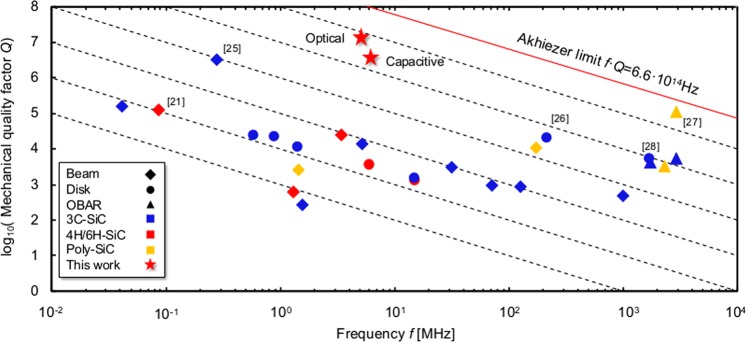
Breaking through the barrier of monocrystalline 4H-SiC resonators with ultra-high *Q*-factors. To date, other SiC resonator designs have shown *Q*-factors below 200 k^21^ with the notable exception of highly-stressed 3C-SiC beam resonators which have shown *Q*s near 3 M at 278 kHz^25^, corresponding to *f* · *Q* = 8.3 · 10^11^ Hz. Polycrystalline SiC OBARs display high *f* · *Q* products in the gigahertz frequency range, a range unsuitable for resonant gyroscope applications. Dotted lines denote constant *f* · *Q* products from 10^9^ Hz to 10^14^ Hz. Overtone Bulk Acoustic Resonators (OBARs)^26–28^.

The details of the apparatus to measure optically-transduced SiC BAW disk resonators are given in the Methods section. The measurements of 4 mm-diameter optical SiC disk resonators reveal *Q*-factors as high as 18 M at 5.3 MHz for the m = 8 elliptical modes (Fig. 3d,e), close to the simulated surface TED limit as well as as-born m = 9 frequency splits as small as 3 ppm (Fig. 3f). These larger SiC BAW disk resonators exhibit an astonishing *f* · *Q* = 9.5 · 10^13^ Hz, which is approximately 5-fold over the absolute quantum limit of Si. Moreover, all the in-plane elliptical modes from 2 MHz to 7 MHz can be measured with this apparatus and compared with simulations. Figure 3e displays the effectiveness of the enhanced PnC for modes which satisfy the frequency requirements of the 2.5 D substrate-decoupling approach. Multiple modes exhibit *Q*s beyond 1 M; however, *Q*s beyond 10 M are achieved only by modes with frequencies in the PnC bandgap and quarter-wavelength matched with the Si handle layer. These measurements validate that quarter-wavelength-matching sets stringent requirements; the acoustic properties and the thickness of the Si handle layer set the optimal resonance frequency of SiC micromechanical resonators to exhibit ultra-low dissipation. For the first time, *Q*s beyond the reach of Si have been demonstrated in SiC micromechanical resonators. The extraordinary low intrinsic losses of monocrystalline SiC may be revealed and show its advantages in BAW resonators that are completely substrate-decoupled, operating in the deep adiabatic regime and possess deep sub-micron level roughness, breaking through the barriers in prior efforts on SiC resonators (Fig. 4).

## Discussion

SiC is a promising material for integrated micromechanical resonators and resonant gyroscopes with ultra-low dissipation. The measurement results suggest centrally-supported and perforation-free 4H-SiC BAW disk resonators are ideally poised to support gyroscopic modes with ultra-high *Q* beyond the range available to Si. Measurements validate that the 2.5 D non-coplanar substrate decoupling approach is sensitive to frequency inaccuracies (see Supplementary Note 1). Modes with frequencies that do not fulfill the quarter-wavelength-matching requirements or that are not within the acoustic bandgap of the PnC consistently exhibit low *Q*s^14^, showing that both conditions must be met simultaneously to completely circumvent anchor loss in centrally-supported and perforation-free 4H-SiC BAW disk resonators with ultra-high *Q*-factors limited by quantum loss mechanisms. Extrapolation based on TED simulations and on measurements of both optical and capacitive disks reveal the gyroscopic m = 3 modes of the 6.29 MHz SiC BAW disk resonators may achieve *Q*s beyond 25 M if the sidewall roughness is trimmed from 2.5 µm to below 500 nm. Achieving even higher *Q*s to enable navigation-grade SiC gyroscopes will require pushing the m = 3 modes further in the adiabatic regime. For example, the *Q*-factor of an m = 3 mode at 2.5 MHz will be surface TED limited to 100 M assuming 500 nm-wide striations.

Akin to (100) Si, n-doped monocrystalline on-axis 4H-SiC substrates support degenerate BAW gyroscopic m = 3 modes in disk resonators. As demonstrated in this paper, the m = 3 elliptical modes implemented in 4H-SiC exhibit both ultra-high *Q*s near 3.8 M and low as-born frequency splits Δ*f*/*f* near 13 ppm. The measurement of higher *Q*s beyond 18 M and smaller frequency splits near 3 ppm in optically-interrogated disks suggest SiC resonant BAW gyroscopes may demonstrate sensitivities unknown to their (100) Si counterparts. Bulk micromachining with nanoscale precision is a critical step in demonstrating SiC gyroscopes with ultra-low dissipation. Etching SiC trenches with nanoscale smoothness is required both to implement advanced deep reactive ion etching processes compatible with a modified version of the HARPSS process but also to reach the Akhiezer limit of SiC and take the full advantage of the material capability. Advanced bulk micromachining processing often involve high temperature steps to anneal, oxidize or deposit materials such as polySi (see Supplementary Note 2). The CTE-mismatch between SiC and Si limit the thermal budget; temperatures greater than 900 °C warp the SiCOI substrates, making these additional processing steps challenging to implement. Current efforts aim to reduce the thermal budget of these processing steps or to manufacture SiC-on-SiC substrates, free of CTE-mismatch issues. With evidence of ultra-high *Q*-factors exceeding 18 M, electrostatic transduction, and as-fabricated frequency splits as low as 3 ppm, 4H-SiC is a promising substrate for robust BAW gyroscopes with ultra-high sensitivity and stability for high-end industrial and navigation applications.

## Methods

### Test apparatus to measure electrostatically-transduced and centrally-supported SiC BAW disk resonators

A schematic of the test apparatus is shown in Fig. 1e. The capacitive SiC BAW disk resonators are wire bonded to a simple PCB board with transimpedance amplifiers (TIAs) and placed in a vacuum chamber with Torr levels pressure. Due to the high stiffness of BAW modes, air damping is negligible below 100 mTorr, a pressure range greatly exceeding our pressure set-point. After 30 minutes of settling time to allow the device and PCB board to reach thermal equilibrium, the resonators are biased at 25V_p_ and their frequency characteristics are recorded with a network analyzer that is calibrated to remove feedthrough. There are 12 electrodes that surround the SiC disks: 3 pairs of electrodes, coinciding with the maximum deformation of one of the m = 3 modes are used to actuate and readout the motion of that mode, while the remaining 3 pairs are used to drive and sense the other m = 3 mode with a mode shape orthogonal to the first m = 3 mode.

The characteristics of electrostatically-transduced disks are as follow: SiC device layer thickness: 55 μm, Si handle layer thickness: 380 μm, disk diameter: 1440 μm, pedestal diameter to disk diameter ratio: near 5%, BOX thickness: 6 μm, transduction gap width: 4.2 μm, maximum surface roughness: 1–2 μm, mode of interest: m = 3 in-plane elliptical modes, resonance frequency: 6.29 MHz, maximum *Q*: 3.8 M corresponding to *f* · *Q* = 2.4 · 10^13^ Hz, minimum frequency split Δ*f*/*f*: 13 ppm.

### Test apparatus to measure optically-transduced and centrally-supported SiC BAW disk resonators

A schematic of the test apparatus is shown in Fig. 1f. The die containing the optically-transduced 4 mm wide SiC BAW disk resonators is glued with crystal bond to a 5 MHz shear piezoelectric actuator from Boston Piezo-Optics^®^ and is placed in a vacuum chamber with optical and electrical access. The die is glued to a 5 MHz piezoelectric shear actuator to facilitate the transfer of energy from the actuator to the die. A firm and practically permanent adhesion is mandated by the 5 MHz resonance frequency. The piezoelectric actuators are controlled by an Agilent Network^®^ analyzer via an RF amplifier. The motion of the disks is sensed by a 1D laser Doppler vibrometer (LDV) from Polytec^®^ using a 20X magnification lens. The LDV is placed on an xyz stage with micrometric control to precisely align the laser head with the sidewall of the SiC disk. The glass window allowing for optical access is optically-rated to minimize absorption at the laser’s wavelength (633 nm). Further, the direction of the LDV head is about 5° off the normal direction of the glass window to prevent receiving undesirable reflected light.

The characteristics of optically-interrogated SiC BAW disks are as follow: SiC device layer thickness: 100 μm, Si handle layer thickness: 450 µm, disk diameter: 4 mm, pedestal diameter to disk diameter ratio: 5%, BOX thickness: 4 μm, maximum surface roughness: 500 nm, mode of interest: m = 8 and m = 9 in-plane elliptical modes, resonance frequency: 5.3 MHz, maximum *Q*: 18 M corresponding to *f* · *Q* = 9.5 · 10^13^ Hz, minimum frequency split Δ*f*/*f*: 3 ppm for the m = 9 mode.

## Supplementary information

Supplementary Information
