# Supplementary material for: Monocrystalline Silicon Carbide Disk Resonators on Phononic Crystals with Ultra-Low Dissipation Bulk Acoustic Wave Modes
Source: Sci Rep. 2019 Dec 10;9:18698. doi: 10.1038/s41598-019-54278-9 (PMC6904713; doi:10.1038/s41598-019-54278-9)
Supplement: Supplementary file 1 — Supplementary Information [file 41598_2019_54278_MOESM1_ESM.pdf]

Monocrystalline Silicon Carbide Disk Resonators on Phononic Crystals with Ultra-Low Dissipation Bulk Acoustic Wave Modes

Benoit Hamelin[1*], Jeremy Yang [2], Anosh Daruwalla[1], Haoran Wen[1] & Farrokh Ayazi[1]

Supplementary information

Supplementary Note 1: Accurate numerical simulations of 4H-SiC disk resonators

With a greater generalized Zener ratio (Z=12), 3C-SiC is more prone to anchor loss than monocrystalline 4H-SiC (Z=3.4)[1,2], making 4H-SiC a preferred SiC polytype in the quest of ultra-low dissipation. Despite availability of 6" production-grade monocrystalline 4H-SiC substrates, the reported elastic constants of 4H-SiC differ considerably, particularly for shear constants with measurements varying by over 7%. The average and standard deviation of reported measurements of elastic constants of 4H-SiC are: C_{11}=508±14GPa, C_{12}=96±GPa, C_{13}=52±3GPa, C_{33}=550±8GPa, C_{44}=164±6GPa, C_{66}=206±7GPa.[3–7]. In this work, anchor loss is circumvented in part by matching the resonance frequency of the SiC BAW disk resonator to the quarter-wavelength of the Si handle layer and a near-7% inaccuracy of the resonance frequency is sufficient to degrade Q_{ANCHOR} from near 1B to 10-100M. To circumvent this knowledge gap, we have fabricated two disparate batches of SiC disks. The first fabrication batch enabled the measurement of a repeatable 1.06 multiplicative factor between fabricated and simulated resonance frequencies for all the elliptical modes up to 7MHz, with the exception of the radial m=0 mode which frequency is accurately predicted. The frequency accuracy of the radial mode suggests that the reported value of C_{11} is accurate. Additional resonators should be fabricated to accurately measure the other elastic constants and their sensitivity to temperature, to enable accurate designing of on-axis 4H-SiCOI resonators. The second fabrication batch took into account the 6% frequency error; in this work, fabricated resonators are 0.6% of their target frequency, sufficient to ensure the efficiency of 2.5D substrate-decoupling approach.

Supplementary Note 2: Wafer-level bulk-micromachining of on-axis monocrystalline 4H-SiCOI

The introduction of fusion-bonded silicon-on-insulator (SOIs) substrates has been transformative, allowing for bulk micro-machining with nanoscale-precision of Si MEMS devices using the Bosch process and other advanced process technologies including HARPSS[8]. While production-grade 4" and 6" SiC substrates are now commercially available, the counterpart SiCOIs are not. In addition, the field of SiC deep reactive ion etching (DRIE) is also nascent and far from the maturity of Si DRIE[9]. Consequently, the literature on DRIE of high-aspect-ratio trenches in thick monocrystalline SiCOI substrates is extremely scarce[10]. In this work, n-doped 4H-SiCOI substrates are demonstrated using 100mm on-axis (Cree®) and 150mm 4°-off-axis (II-VI®). Like commercially-available SOIs, the 4H-SiCOI substrates are formed by fusion bonding thermally-oxidized SiC and Si wafers followed by wafer-level thinning. The 500µm-thick 4" SiC wafers are thoroughly cleaned in Piranha and SC-1, dried, and thermally oxidized for 12 hours to grow a pristine 1.5µm thick SiO_2 layer on the C-face of the SiC wafers. Following the same process, a 3µm thick SiO_2 layer is thermally grown on the Si wafers. The wafers are polished to achieve a surface roughness below 0.5nm (Entrepix®). The wafers are then brought in close proximity in vacuum and a slight force is applied at the center of the wafer stack (EVG®). As the pressure wave travels from the center towards the edges, residual air is expelled and the wafers bond. Because of their curved edges, Si and SiC do not bond 5mm from their edges[11]. To strengthen the SiO_2-SiO_2 bond, the SiCOI wafers are annealed at 400°C for 2 hours. It is important to note that wafer level annealing at higher temperature may lead to severe wafer bowing due to the CTE mismatch between Si and SiC. The wafers are then either lapped (Entrepix®) or grinded (Disco®) with total thickness variations on the order of 1-3µm. For the rest of the fabrication process, wafer-level high-temperature processing steps are avoided. The first fabrication step consists in defining the polySi plug by DRIE of the SiC layer and of the buried oxide (BOX) layer. A 5µm-thick Ni hard mask is electroplated on a Ti/Cu (20/100nm) seed layer and is used as a hard mask. The seed layers and photoresist are respectively removed by a mixture of acetic acid, hydrogen peroxide and deionized water in the volumetric ratio 1:1:10 for 30s at room temperature and a minute

[1]Department of Electrical and Computer Engineering, Georgia Institute of Technology, 777 Atlantic Drive NW, Atlanta, GA 30332, USA, [2]School of Physics, Georgia Institute of Technology, 837 State Street, Atlanta, GA 30332, USA [*] Correspondence and request for material should be addressed to B.H. (email: hamelin@gatech.edu)

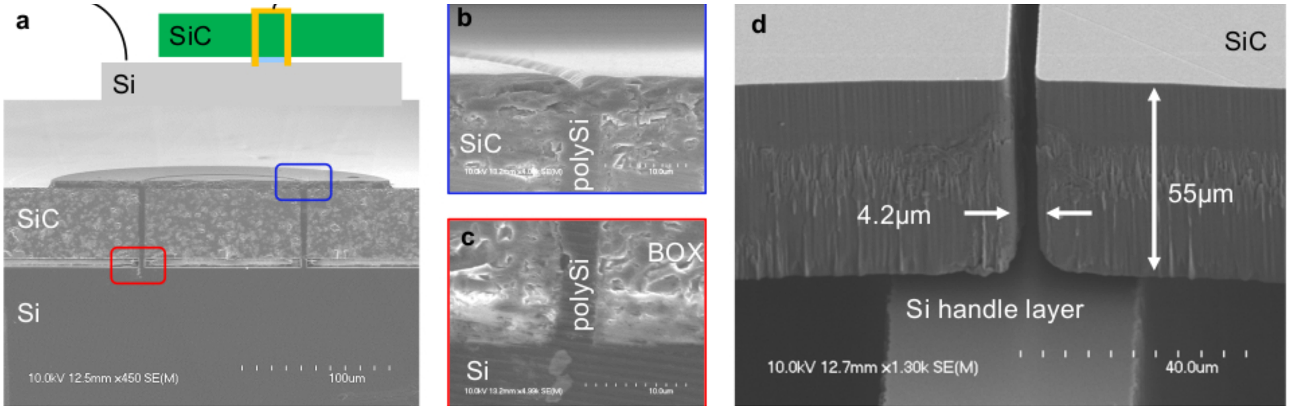

Figure S1. Advanced bulk micromachining of SiCOI substrates. (a) Polycrystalline silicon-refilled SiC trenches etched through the BOX layer and into the Si handle layer create an electrical path (see inset) towards the SiC disk and precisely define the SiO_2 pedestal during HF releasing. (b and c) The trenches are completely refilled, free of voids, and fully protect the SiO_2 pedestal during wet HF releasing. (d) A SiC dry etching recipe has been developed to minimize footing at the BOX/SiC interface, but has created vertical striations, which must be suppressed to reach higher Q-factors in capacitive SiC disk resonators. The vertical striations along the SiC sidewall are at most 2.5µm wide and load Q by introducing new thermo-mechanical couplings. Scale bars in a,b,c, and d: 100µm, 10µm, 10µm, 40µm

long O_2 plasma exposure in an Advanced Vision RIE. The SiC trenches are etched in an STS AOE etcher with nano- and microscale roughness ranging from 50nm to 2.5µm. To ensure proper electrical connection, the trenches are also etched through the BOX layer; the trenches land in the Si handle layer. The polySi-refilled trenches are free of any voids and electrically connect the SiC device layer to the Si handle layer (Figures S1.a-c). The SiC electrodes are covered with polySi. This is necessary because Al wire bonds do not form an ohmic contact with n-doped SiC at room temperature. In this work, polySi is preferred over various metals (e.g. Ni) for its forward-compatibility with advanced DRIE processing, including a modified version of HARPSS[8] applied to SiCOI substrates. The SiC trenches are designed to land smoothly on the BOX layer. The SiC DRIE recipe has been modified to improve the selectivity to SiO_2 at the cost of increasing the sidewall's roughness (Figure S1.d). Future optimization will manage a better balance between high SiC etch rate, vertical and smooth sidewalls, and landing on the BOX layer without generating sidewall damage in the form of roughness and footing. The PnC occupies as much area in the Si handle layers as possible to make it more forgiving to design and fabrication inaccuracies[12]. A honeycomb unit cell is used in this work because it generates a larger acoustic bandgap than other implementations with comparable filling factors β, lessening fabrication requirements to achieve similar Q-factors. Finally, the 6.5mm by 8mm dies are individually diced (Figure **??**), released in HF and annealed twice in an RTP at 1,200°C for two minutes. To prevent oxidation of the polySi layer on top of the SiC electrodes, the RTP chamber is filled with N_2. Noteworthy, flowing an excessive amount of N_2 at 1,200°C can result in a damaging nitridation of the polySi thin-film. Finally, annealing at lower temperatures such as 1,100°C has proven insufficient to form an ohmic contact between SiC and polySi. Under optimal conditions, electrical resistance between 10 and 500Ω are repeatedly measured between n-doped SiC and in-situ n-doped polySi.

References

1. Lethbridge, Z. A., Walton, R. I., Marmier, A. S., Smith, C. W. & Evans, K. E. Elastic anisotropy and extreme Poisson's ratios in single crystals. *Acta Mater.* **58**, 6444–6451, DOI: http://dx.doi.org/10.1016/j.actamat.2010.08.006 (2010).
2. Hao, Z. & Ayazi, F. Support loss in the radial bulk-mode vibrations of center-supported micromechanical disk resonators. *Sensors Actuators, A Phys.* **134**, 582–593, DOI: http://dx.doi.org/10.1016/j.sna.2006.05.020 (2007).
3. Kamitani, K. *et al.* The elastic constants of silicon carbide: A Brillouin-scattering study of 4H and 6H SiC single crystals. *J. Appl. Phys.* **82**, 3152–3154, DOI: http://dx.doi.org/10.1063/1.366100 (1997).
4. Mirgorodsky, A. P., Smirnov, M. B., Abdelmounîm, E., Merle, T. & Quintard, P. E. Molecular approach to the modeling of elasticity and piezoelectricity of SiC polytypes. *Phys. Rev. B* **52**, 3993–4000, DOI: http://dx.doi.org/10.1103/PhysRevB.52.3993 (1995).
5. Nuruzzaman, M., Islam, M. A., Alam, M. A., Shah, M. A. H. & Karim, A. M. M. T. Structural , elastic and electronic properties of 2H- and 4H-SiC. *Int. J. Eng. Res. Appl.* **5**, 48–52 (2015).

6. Karmann, S., Helbig, R. & Stein, R. A. Piezoelectric properties and elastic constants of 4H and 6H SiC at temperatures 4-320 K. *J. Appl. Phys.* **66**, 3922–3924, DOI: http://dx.doi.org/10.1063/1.344477 (1989).

7. Li, Z. & Bradt, R. C. The single crystal elastic constants of hexagonal SiC to 1000°C. *Int. J. High Technol. Ceram.* **4**, 1–10, DOI: http://dx.doi.org/10.1016/0267-3762(88)90060-4 (1988).

8. Ayazi, F. & Najafi, K. High Aspect-Ratio Combined Poly and Single-Crystal. *J. Micromechanical Syst.* **9**, 288–294, DOI: http://dx.doi.org/10.1109/84.870053 (2000).

9. Dowling, K. M., Ransom, E. H. & Senesky, D. G. Profile evolution of high aspect ratio silicon carbide trenches by inductive coupled plasma etching. *J. Microelectromechanical Syst.* **26**, 135–142, DOI: http://dx.doi.org/10.1109/JMEMS.2016.2621131 (2017).

10. Luna, L. *et al.* SiC Wafer Bonding and Deep Reactive Ion Etching Towards High-Aspect Ratio SiC MEMS Fabrication. *ECS Trans.* **86**, 105–110, DOI: http://dx.doi.org/10.1149/08605.0105ecst (2018).

11. Ko, S.-D., Hamelin, B., Yang, J. & Ayazi, F. High-Q monocrystalline silicon carbide disk resonators fabricated using DRIE of thick SiC-on-insulator substrates. In *Proc. IEEE Int. Conf. Micro Electro Mech. Syst.*, DOI: http://dx.doi.org/10.1109/MEMSYS.2018.8346726 (2018).

12. Yang, J., Hamelin, B., Ko, S. & Ayazi, F. Ultra-High Q Monocrystalline Silicon Carbide disk Resonators Anchored Upon a Phononic Crystal. In *2018 Solid State Sensor, Actuator Microsystems Work.*, 83–86, DOI: http://dx.doi.org/10.31438/trf.hh2018.22 (2018).
